# Rumination impairs the control of stimulus-induced retrieval of irrelevant information, but not attention, control, or response selection in general

**DOI:** 10.1007/s00426-018-0986-7

**Published:** 2018-01-23

**Authors:** Lorenza S. Colzato, Laura Steenbergen, Bernhard Hommel

**Affiliations:** 1 Cognitive Psychology Unit and Leiden Institute for Brain and Cognition, Leiden University, Leiden, The Netherlands; 2 Department of Cognitive Psychology, Institute of Cognitive Neuroscience, Faculty of Psychology, Ruhr University Bochum, Bochum, Germany; 3 Institute for Sports and Sport Science, University of Kassel, Kassel, Germany; 4 Amsterdam Brain and Cognition (ABC), University of Amsterdam, Amsterdam, The Netherlands; 5 Cognitive Psychology Unit, Leiden University Institute for Psychological Research, Wassenaarseweg 52, 2333 AK Leiden, The Netherlands

## Abstract

The aim of the study was to throw more light on the relationship between rumination and cognitive-control processes. Seventy-eight adults were assessed with respect to rumination tendencies by means of the LEIDS-r before performing a Stroop task, an event-file task assessing the automatic retrieval of irrelevant information, an attentional set-shifting task, and the Attentional Network Task, which provided scores for alerting, orienting, and executive control functioning. The size of the Stroop effect and irrelevant retrieval in the event-five task were positively correlated with the tendency to ruminate, while all other scores did not correlate with any rumination scale. Controlling for depressive tendencies eliminated the Stroop-related finding (an observation that may account for previous failures to replicate), but not the event-file finding. Taken altogether, our results suggest that rumination does not affect attention, executive control, or response selection in general, but rather selectively impairs the control of stimulus-induced retrieval of irrelevant information.

## Introduction

Adaptive human behavior is commonly taken to reflect the operation of cognitive-control processes that organize lower-level information-processing streams according to the current task and intention. Increasing evidence suggests that individuals exhibit systematic differences in the way they perform cognitive-control processes, and it was these differences that the present study was aimed at. In particular, people have shown systematic interindividual and intraindividual differences regarding the degree to which their performance reflects cognitive persistence and flexibility (Hommel, 2015; Hommel & Colzato, 2017a, b). Persistence and flexibility have been considered two antagonistic metacontrol strategies (i.e., strategies that control cognitive control; Goschke, 2003; Cools & d’Esposito, 2011) that can be considered as the extreme poles of a common metacontrol dimension (Hommel, 2015). Changing tasks and environmental conditions require continuous readjustments of the balance between persistence and flexibility, which induces intraindividual variability (Akbari Chermahini & Hommel, 2010; Dreisbach & Goschke, 2004; Herd et al., 2014; Müller et al., 2007), and people differ systematically with respect to the efficiency of the degree to which this balance can be achieved (Arbula, Capizzi, Lombardo, & Vallesi, 2016; Babcock & Vallesi, 2017; for a review, see Hommel & Colzato, 2017b).

The goal of this study was to investigate a possibly potent personality characteristic that might be associated with a systematic metacontrol bias, at least with respect to some control-relevant tasks: rumination. According to Whitmer and Gotlib (2013, p. 1036), “rumination is generally defined as repetitive thinking about negative personal concerns and/or about the implications, causes, and meanings of a negative mood”. In terms of metacontrol theory, this amounts to a strong bias toward persistence, at the expense of flexibility. Rumination and cognitive reactivity to sad mood in general has important clinical implications, and the degree and amount of rumination are informative regarding the probability of becoming chronically depressed (Beck, 1967; Nolen-Hoeksema, Morrow, & Fredrickson, 1993; Kuehner & Weber, 1999; Nolen-Hoeksema, 2000; Spasojevic & Alloy, 2001; Moulds et al., 2008). However, the relevance of rumination is not restricted to clinical cases, more so as many healthy individuals tend to ruminate at least occasionally. From a metacontrol perspective, it is interesting to consider that individuals who tend to ruminate show difficulties in disengaging from or forgetting information that is no longer relevant (Whitmer & Gotli, 2013), which has been shown in studies with healthy individuals. For instance, the personal tendency to ruminate was positively correlated with bigger struggle in disengaging from no-longer-relevant information, but not in ignoring external distracters, in a modified Sternberg task (Joormann & Gotlib, 2010), and with greater difficulty to ignore irrelevant information in a Stroop task (Philippot and Brutoux, 2008). Unfortunately, however, the overall picture is disturbed by at least partial non-replication. For instance, other studies found Stroop performance to be better (Altamirano, Miyake & Whitmer, 2010) or unrelated to rumination in samples of dysphoric and nondysphoric participants (Krompinger and Simons, 2011 and Meiran, Diamond, Toder, and Nemets, 2011). On the one hand, this may raise serious questions regarding replicability but, on the other, differences in task and design cannot be ruled out as possible factors.

Given the present state of affairs, we were interested in testing the impact of rumination in healthy participants on a broader set of control-related tasks. In addition to various demographic indicators, we assessed rumination and cognitive reactivity to transient changes in sad mood by means of the revised Leiden Index of Depression Sensitivity (LEIDS-r; van der Does & Williams, 2003). We studied the impact of LEIDS-r scores on four tasks or task-performance indicators. The first choice was obviously the Stroop task, as this has been used in various rumination studies previously. Given previous observations, the question was whether we could replicate the observation of a positive correlation between rumination tendency and the inability to suppress irrelevant information (as indicated by the size of the Stroop effect).

The second was the event-file task developed by Hommel (1998). The task assesses the degree to which specific combinations (bindings) of stimulus and response features are automatically retrieved in the next trial. Previous studies have shown that individuals with suboptimal top-down cognitive-control abilities, such as children and elderly (Hommel, Kray & Lindenberger, 2011), people low in fluid intelligence (Colzato, van Wouwe, Lavender & Hommel, 2006), or individuals suffering from autistic spectrum disorders (Zmigrod et al., 2013), are more likely to retrieve (or less likely to suppress) bindings between irrelevant stimulus features and the response. At the same time, neurofeedback-based cognitive-control training was able to eliminate the retrieval of these bindings, in addition to increasing the intelligence score (Keizer, Verment & Hommel, 2010; Keizer, Verschoor, Verment & Hommel, 2010). This suggests that the degree to which bindings between irrelevant stimulus features and the response formed in the previous trial affects performance in the present trial represents the degree to which people can control binding retrieval. If so, more rumination should be associated with more pronounced effects of bindings between irrelevant stimulus features and the response.

The Stroop and the event-file task tap into the efficiency of handling of memory information that is activated by the current stimulus. A more pronounced effect of task-irrelevant information would thus indicate a lack of selectivity with respect to the information that the present stimulus ought to (re-)activate, with the result that falsely (re-)activated memory traces compete with task-relevant information for selection. A related, but somewhat different aspect of information-processing efficiency is assessed by an attentional set-shifting task developed by Dreisbach and Goschke (2004). Participants usually perform a letter or digit classification task and the performance is measured before and after the switch to a different version of the task, so that the difference represents set-switching costs. In a “perseveration” condition, participants switch to target stimuli in a novel color and try to ignore the previous target, which continues to be present. Switching costs in this condition are compared with switching costs in a “learned irrelevance” condition, where participants switch to target stimuli in a previously ignored color, while ignoring the previously relevant color. Individual differences related to persistence and flexibility should affect performance in the two conditions differently. A lack of persistence or a bias toward flexibility should support switching in the perseveration condition, but impair switching in the learned irrelevance condition, as flexibility should bias attention toward novel stimuli. Accordingly, one would expect the opposite pattern in individuals with a strong rumination tendency: they would have a hard time turning to something novel and thus show relatively poor performance in the perseveration condition, but relatively good performance in the learned irrelevance condition. Given that the attentional set-shifting task shares the characteristic of the Stroop in the event-file task of tapping into memory control, but focuses more on the impact of past attentional settings on present attentional control (a factor that can be suspected to affect Stroop and switching performance differently: Herd et al., 2014), we decided to also include it as the third task in the present study.

Note that both the Stroop task and the event-file task assess selectivity in handling stimulus-induced activations of internal information. A Stroop effect can only be obtained if the meaning of the color word is retrieved to a degree that it interferes with the determination of the stimulus color. Hence, the size of the Stroop effect should depend on retrieval control, just as assessed by the event-file task. However, the recent emphasis on attentional processes in the context of rumination (Whitmer & Gotlib, 2013) suggests that more direct assessments of attention to external information might also be of interest. The most comprehensive assessment in this respect is provided by the Attentional Network Task (ANT) developed by Fan et al. (2002). This task is a hybrid that combines Posner’s (1980) cued reaction time (RT) task and Eriksen and Eriksen’s (1974) flanker task. It provides three indicators that are assumed to reflect the efficiency of the alerting network (i.e., preparation for a stimulus event), the orienting network [i.e., (re-)allocating visual attention to a new stimulus event], and the executive-control network (i.e., resolving stimulus-induced response-selection conflict). Note that none of these measures tap into retrieval control, which is an issue that will be important for interpreting our findings.

As the ANT has not yet been used to investigate the impact of rumination or to study individual differences in metacontrol, different predictions are possible. Given the emphasis of recent rumination research on attentional control, one would expect a systematic association between the personal tendency to ruminate and performance on all three indicators of the ANT task. Hence, alertness, orientation ability, and response-conflict resolution should be impaired more the more people tend to ruminate. Another possibility is that rumination is more selectively related to more central processes, with response selection being an obvious choice (Johnston, McCann & Remington, 1995). If so, it is possible that rumination only affects tasks or task indicators that induce response conflict, such as the Stroop, the event-file task, the attentional set-shifting task, and the executive-control indicator of the ANT. Finally, it is interesting to note that the previous metacontrol studies did not show any systematic impact of individual metacontrol policies on the Simon effect (Colzato, Sellaro, Samara, & Hommel, 2015), even though this effect is commonly taken to reflect the same kind of response conflict that is induced by the flanker task. Accordingly, it might also be possible that rumination is even more selectively related to online retrieval control, in which case the correlation might be restricted to the Stroop and the event-file task.

## Method

### Participants

Ninety adults participated in a multi-session multivariate study on individual predictors of basic cognitive, social, and sensorimotor processes. After excluding participants who missed multiple test sessions (*n* = 12), the working sample consisted of 78 Leiden University healthy undergraduate students. The demographic statistics are provided in Table 1. Participants were recruited via an online recruiting system and were offered either course credits or a financial reward for their participation. Once recruited, all participants were screened individually using the Mini International Neuropsychiatric Interview (M.I.N.I.; Sheehan et al., 1998). The M.I.N.I. is a short, structured interview of about 15 min that screens for several psychiatric disorders and drug use, often used in clinical and cognitive neuroscience research (Colzato, van den Wildenberg & Hommel, 2014; Colzato, Pratt & Hommel, 2010; Sheehan et al., 1998). Participants with no psychiatric or neurological disorders and no personal or family history of depression or migraine were considered suitable to take part in the study. The study conformed with the ethical standards of the Declaration of Helsinki and the protocol was approved by the local ethical committee (Leiden University, Institute for Psychological Research). Written informed consent was obtained from all participants.

**Table 1:** Demographic characteristics, descriptive statistics for mean scores, and standard deviations (shown in parentheses) on the LEIDS-R and BDI-II

Variables (SD)	
*N* (M:F)	78 (38:40)
Age	22.48 (3.74)
Body mass index	25.01 (4.04)
Physical activity hours per day^a^	2.84 (2.51)
LEIDS-R	
Aggression	7.11 (3.86)
Control	8.28 (3.12)
Hopelessness	3.38 (2.95)
Risk aversion	9.40 (3.69)
Rumination	10.74 (4.33)
Acceptance	2.61 (2.36)
Total	41.53 (12.96)
BDI-II	
Total	6.13 (4.58)

^a^Transportation by bike included (favorite mode of transportation by Dutch students)

### Experimental tasks and procedure

The tasks selected to investigate the relation of stimulus–response conflict cost and cognitive reactivity to sad mood (as indexed by LEIDS-r) were the Stroop task, the event-file task, the attentional set-shifting task and the ANT. To control for depressive symptoms, participants filled in the BDI-II. The behavioral tasks and questionnaires were administered in five separate sessions with at least 24 h in between to avoid any carryover effects of one task and/or questionnaire to the others. Before completion of each task, participants were asked to rate their mood on a 9 × 9 Pleasure × Arousal grid (Russell, Weiss, & Mendelsohn, 1989), with values ranging from − 4 to 4 (“Footnote 1”).

#### LEIDS-R

The LEIDS-r (van der Does & Williams, 2003) is a self-report questionnaire consisting of 34 items that assesses to what extent dysfunctional thoughts are activated when experiencing mild dysphoria (i.e., it measures cognitive reactivity to sad mood, also referred to as vulnerability to depression). LEIDS-r scores have been found to predict depression incidence in multiple longitudinal studies and to correlate with depression risk factors, such as depression history (Moulds et al., 2008), genetic markers of depression (Antypa & van der Does, 2010), and reaction to tryptophan depletion (Booij & van der Does, 2007). Following Steenbergen et al. (2015), before responding to the items, participants were requested to take a few minutes to imagine how they would feel and think if they were to experience a sad mood and then to specify, on a 5-point Likert scale ranging from 0 (i.e., ‘not at all’) to 4 (‘very strongly’), the extent to which each statement applied to them. It was emphasized that the statements applied to the situations when “it is certainly not a good day, but you don’t feel truly down or depressed”. The scale consists of six subscales that measure vulnerability with respect to:

- aggression (e.g., When I feel down, I lose my temper more easily);
- hopelessness/suicidality (e.g., When I feel down, I more often feel hopeless about everything; When I feel sad, I feel more that people would be better off if I were dead);
- acceptance/coping (e.g., When I am sad, I feel more like myself);
- control/perfectionism (e.g., I work harder when I feel down);
- risk aversion (e.g., When I feel down, I take fewer risks);
- rumination (e.g., When I feel sad, I more often think about how my life could have been different).

Hopelessness and acceptance/coping both consist of five items, with a maximum score of 20 per subscale, whereas the other scales comprise six items with a maximum score of 24 per subscale. The LEIDS-r total score is calculated by adding up the scores from each subscale, resulting in total scores ranging from 0 to 136. Internal consistency (Cronbach’s alpha; *α*) is 0.89 for the LEIDS total score and ranges between 0.62 (acceptance/coping) and 0.84 for the subscales (hopelessness/suicidality; Antypa & van der Does, 2010; Williams, van der Does, Barnhofer, Craner & Segal, 2008). Table 1 gives a summary of the scores on the LEIDS-R.

#### BDI-II

The BDI-II (Beck, Steer, Ball & Ranieri, 1996) is a widely used 21-item multiple-choice self-report questionnaire with high internal consistency (*α* = 0.91; Beck et al., 1996), which assesses the existence and severity of current (past 2 weeks) depressive symptoms. The study used the Dutch translation validated by Van der Does (2002b). The BDI-II has been found to be a valid indicator of depression and shows good diagnostic discrimination (Dozois, Dobson & Ahnberg, 1998). Participants were presented with items related to symptoms of depression and asked to choose for each item the statement that better described how they have been feeling during the past 2 weeks (including the current day). Items are rated on a 4-point scale ranging from 0 to 3 in terms of severity. The total score is calculated by adding up all items; hence, the scores range between 0 and 63 (0–13: minimal depression, 14–19: mild depression, 20–28: moderate depression and 29–63: severe depression; van der Does, 2002a). Table 1 gives a summary of the scores on the BDI-II. Participants did not show any sign of depression: only minimal/mild scores were observed.

#### Stroop task

We used a manual, computerized variant of the original Stroop task (Stroop, 1935), adapted from Gehring and Fencsik (2001). A fixation cross was displayed on the computer monitor 500 ms before the stimulus appeared and until the participant responded. Stimulus words were the Dutch color names for ‘yellow’, ‘blue’, ‘green’, and ‘red’ (‘geel’, ‘blauw’, ‘groen’, and ‘rood’, respectively). Stimuli appeared on the screen printed in one of those four colors. The word and color were chosen at random on each trial from a list of 40 incompatible and 40 compatible stimuli. Participants were instructed to respond to the color of the stimulus word and ignore the word itself, and react as fast and accurately as possible. Responses were made by pressing the following buttons of the QWERTY keyboard: “z” for yellow, “x” for blue, “>” for green, and “?” for red. Participants completed one block of 20 practice trials, in which the words were replaced by ‘xxxxx’ to get used to the button–color combinations. After this, four blocks followed, each consisting of 80 trials. The task took 10 min to complete.

#### Event-file task

The task, originally developed by Hommel (1998), was adapted from Colzato et al. (2012). During the task, participants were seated approximately 50 cm from a 17-in. monitor (96 dpi with a refresh rate of 120 Hz). The E-Prime 2.0 software system (Psychology Software Tools, Inc., Pittsburgh, PA, USA) was used to generate the task and collect the responses.

The task measures binding-related aftereffects by examining partial-repetition costs related to combinations of stimulus features (shape and color in our case) and combinations of stimulus features and the response. To manipulate the repetition vs. alternation of stimulus features and responses, each trial involved a response to the presentation of a prime stimulus (S1 → R1), followed by a response to the presentation of a probe stimulus (S2 → R2). Prime and probe stimuli consisted of yellow- or green-colored images of a banana or an apple. The probe trial required a manual binary-choice response (R2) to the shape of the second stimulus S2 (an apple or a banana). The prime trial required a manual response (R1) to the mere onset of the first stimulus (S1). The correct R1 was signaled by a precue in advance of S1 (through a left- or right-pointing arrowhead), so that S1 and R1 could be varied independently, which was necessary to create orthogonal repetitions and alternations of stimulus shape and response. Given that R2 was signaled by the shape of S2, the shape served as a relevant stimulus feature. Color was manipulated as an irrelevant stimulus feature, which was realized by presenting the apple or banana in green or yellow (see Colzato et al., 2013).

Each trial began with the presentation of an arrowhead (stimulus duration = 1500 ms) that pointed to the left or to the right, and that signaled the response to be given upon the onset of the prime stimulus (S1), which appeared after a 1000 ms interstimulus period. The prime stimulus was presented for 500 ms. Participants were instructed to press the left (“z”) key if the arrowhead preceding the prime stimulus pointed to the left, and the right (“m”) key if the arrowhead pointed to the right. After the response to the prime, the probe stimulus (S2) appeared (stimulus duration = 1500 ms). Participants were instructed to respond to the shape of the stimulus: the presentation of an “apple” required them to press the left (“z”) key, whereas the presentation of a “banana” required them to press the right (“m”) key. Participants were asked to respond as quickly and accurately as possible to both S1 and S2.

The task comprised a practice block of 10 trials and an experimental block of 192 trials, presented in a random order. Experimental trials were equally distributed across eight conditions, resulting in the combinations of stimulus features (shape and color) and responses, which could all either repeat or alternate, thus creating a 2 × 2 × 2-factorial design. For present purposes, two effects were of particular relevance: the interaction between shape repetition/alternation and response repetition/alternation (which implies the retrieval of a binding between a relevant stimulus feature and the response) and the interaction between color repetition/alternation and response repetition/alternation (which implies the retrieval of a binding between an irrelevant stimulus feature and the response).

#### Attentional set-shifting task

The experimental paradigm was adapted from Dreisbach et al. (2005) and Tharp and Pickering (2011). It consisted of four blocks of 60 trials each. In each block, participants performed either a letter or digit classification task, and within each block a single attentional switch occurred (switch condition: perseveration or learned irrelevance).

In each trial of the letter task, two different letters (drawn from A, E, O, U, K, M, R, or S) were presented at the same time, one above the other, in different colors (red, blue, or yellow). At the start of each block, participants were required to react to the letter in a particular color (e.g., red) and indicate whether the target letter was a vowel or a consonant. The nontarget letter acted as a distractor and was presented in one of the non-target colors (e.g., blue). The distractor could either be response compatible (e.g., both target and distractor were vowels) or response incompatible (e.g., distractor = consonant, target = vowel). The target and distractor letters always differed and the target location (above–below) was randomized. After 40 trials, one of two possible attentional switch instructions was given. In the perseveration condition, participants were instructed to respond to the letter in a new color (i.e., yellow), while distractors appeared in the previous target color (i.e., red). In the learned irrelevance condition, participants were required to react to the letter in the previously irrelevant color (i.e., blue), while distractors appeared in the new color (i.e., yellow). Each block consisted of 20 post-switch trials per block.

The digit task (2, 3, 4, 5, 6, 7, 8, and 9) followed the same sequence of events as the letter task, except participants had to react to digits in the target color (olive, purple, or grey) as odd or even. Again, after 40 trials, one of the two possible attentional switch conditions occurred (perseveration/learned irrelevance).

Following Tharp and Pickering (2011), each participant performed each task (letter/digit) once with each switching condition (perseveration/learned irrelevance), resulting in four different experimental blocks, each block consisting of 60 trials (40 pre- and 20 post-switch trials). To minimize the practice effects, the order of the four experimental blocks was counterbalanced such that the same attentional switch was not performed in consecutive blocks. The assignment of stimulus colors was counterbalanced across participants, but remained fixed throughout for each individual participant.

#### Attention network test (ANT)

Participants had to react as fast and accurately as possible to the direction of a central arrow (the target) by pushing the corresponding key (left or right) on the computer keyboard. These targets were preceded by a cue, with four cue type conditions: no cue, center cue (an asterisk substituting the fixation cross), double cue (two asterisks, respectively, appearing above and below the fixation cross), or spatial cue (an asterisk appearing above or below the fixation cross and indexing the location of the upcoming target). Further, flankers were positioned on each side of the target, with three flanker type conditions: two arrows in the same direction as the target (congruent condition), two arrows in the opposite direction of the target (incongruent condition), or two lines (neutral condition). The sequence of each trial was as follows: (1) a central fixation cross (random duration, 400–600 ms); (2) a cue (100 ms); (3) a central fixation cross (400 ms); (4) a target and its flankers, appearing above or below the fixation cross (lasting until the participant responded or for 1700 ms); (5) a central fixation cross [lasting for 3500 ms minus the sum of the first fixation period’s duration and the RT. The ANT consisted of 288 trials, divided in three blocks of 96 trials each (with a short break between blocks). Based on the combination of four cues (no cue, center cue, double cue, spatial cue), three flankers (congruent, incongruent, neutral), two directions of the target arrow (left, right), and two localizations (upper or lower part of the screen), and 48 trial combinations arose. Trials were presented in a random order, and each possible trial was presented twice within a block.

### Statistical analyses

#### Stroop task

RTs (ms) and Percentages of errors (PEs) were assessed for the two conditions: congruent trials (when the word is presented in the same color) and incongruent trials (when the word is presented in a different color). Corrected RTs and PEs were submitted to separate ANOVAs with congruency (congruent vs. incongruent) as within-participant factor. For the RT analysis, we also excluded RTs faster than 100 ms and longer than 2500 ms. The congruency (Stroop) effect was calculated by subtracting the mean RT for congruent trials from the mean RT for incongruent trials. Two participants were eliminated from the analysis for committing more than 25% errors.

#### Event-file task

The retrieval of stimulus–response episodes was assessed by submitting RTs for correct R2 responses and PEs for R2 to separate 2 × 2 ANOVAs with the repetition vs. alternation of response (R1 → R2), stimulus shape and color (S1 → S2) (hereafter referred to as Response, Shape, and Color, respectively) as within-participant factors. For the RT analysis, we also excluded anticipations, that is, RTs faster than 100 ms. Of particular interest were the two-way interactions between repetition effects, as these interactions are diagnostic of the retrieval of a previously created binding of feature combinations (Hommel, 1998). The effect of this retrieval was quantified by calculating the corresponding interaction term, that is, as the difference between RTs for partial repetitions (feature X repeated and feature Y alternated, or vice versa) and the RTs for complete repetitions and alternations. That is, if features X and Y repeated and alternated, their binding effect *B*_XY_ would be calculated as *B*_XY_ = (RT_X/alt,Y/rep_ + RT_X/rep,Y/alt_)/2 − (RT_X/rep,Y/rep_ + RT_X/alt,Y/alt_)/2. A value close to zero means that the repetition effects of the two given features do not interact; a value greater than zero indicates a “binding-type” interaction. In the present study, we focused on the interaction between Color and Response (implying retrieval of the binding between the irrelevant stimulus feature and the response)—our key indicator—and, as a control, the interaction between Shape and Response (implying retrieval of the binding between the relevant stimulus feature of the response).

#### Attentional set-shifting task

Following Dreisbach et al. (2005), for each of the six experimental blocks, the means of the remaining RTs and PEs were computed for consecutive intervals of five trials, separately for response-compatible and response-incompatible trials. The crucial comparison is between the two intervals immediately before the target color switch (Trials 36–40) and immediately after the switch (Trials 41–46). Analyses with larger intervals of ten trials did not substantially change the results. Switch costs were calculated as the difference between post-switch and pre-switch trials for RT and PE, respectively. ANOVA analyses rely on these mean switch costs. Data were merged over the three blocks of each switch condition. This resulted in a 2 (compatibility: compatible vs. incompatible) × 2 (transfer-condition: perseveration vs. learned irrelevance) ANOVA.

#### ANT

Two separate 4 (cue condition: no cue, center cue, double cue, spatial cue) × 3 (flanker type: neutral, congruent, incongruent) ANOVAs on RTs and PEs were carried out. Following Fan et al. (2002), the alerting effect was calculated by subtracting the mean RT of the double-cue conditions from the mean RT of the no-cue conditions; the orienting effect was calculated by subtracting the mean RT of the spatial cue conditions from the mean RT of the center cue; and the executive control effect was calculated by subtracting the mean RT of all congruent flanking conditions, summed across cue types, from the mean RT of incongruent flanking conditions.

#### Spearman–Brown reliability coefficients of cognitive tasks

For the Stroop task, event-file task, and ANT we computed the effects separately from odd- and even-numbered trials. For the attentional set-shifting task, given the limited amount of trials, we computed the effect separately from tasks (digit vs. letter). Then, we computed the correlation between those measures using Spearman–Brown formula.

#### Correlation analyses

The aim of the correlation analysis (Pearson’s correlations coefficients) was to test whether cognitive reactivity to sad mood (as indicated by LEIDS-r total score, and rumination and aggression subscales) interacts with our nine key task indicators (and, as a control, the interaction between stimulus shape and response in the event-file task). We also ran separate correlation analysis (partial correlations) to control for depressive symptoms (BDI-II) and current mood and arousal (affect grid). In addition to standard statistical methods, we also analyzed our data within a Bayesian framework, which allows researchers to quantify and compare the relative likelihood of the data under two competing hypotheses, namely, the alternative (H1) and the null (H0) hypothesis, as indexed by the Bayes factor (Morey & Rouder, 2015; Rouder, Morey, Speckman & Province, 2012). Analyses were performed using JASP 0.8.2.0 software (available on https://jasp-stats.org/). A Bayesian correlational analysis (using the default setting) was carried out to quantify evidence for the presence of a cognitive reactivity to sad mood effect on our nine key task indicators (and, as a control, the interaction between stimulus shape and response in the event-file task).

## Results

### Stroop task

Before starting the Stroop task, the affect grid revealed the following scores: mood (*M* = 1.80; SD = 1.27) and arousal (*M* = 0.03; SD = 1.70). ANOVAs revealed standard Stroop effects in terms of both RTs and PEs: responses were faster and more accurate on congruent (*M* = 758 ms, SD = 141, and *M* = 3.59%, SD = 4.11) than on incongruent trials (*M* = 868 ms, SD = 181, and *M* = 5.49%, SD = 5.09), *F*(1,75) = 199.51, *p* < 0.001, *η*2_p_ = 0.73 (RTs), *F*(1,75) = 27.03, *p* < 0.001, *η*2_p_ = 0.26 (PEs). The resulting Stroop effect was calculated from RTs (incongruent Stroop–congruent Stroop; *M* = 113 ms, SD = 74). SDs in this study were comparable to other studies with bigger sample size (Johnson et al., 2003). Internal reliability of the Stroop effect was very high, (rSB1 = 0.507).

### Event-file task

Before starting the event-file task, the affect grid revealed the following scores: mood (*M* = 1.61; SD = 1.49) and arousal (*M* = 0.11; SD = 1.93). Table 2 provides an overview of the relevant ANOVA outcomes for RTs and PEs obtained for R2. Replicating earlier findings (Hommel 1998; Hommel and Colzato 2004), RTs revealed significant interactions between Shape and Color, *F*(1,77) = 10.16, *p* < 0.01, *η*^2^_p_ = 0.12, between Shape and Response, *F*(1,77) = 186.52, *p* < 0.001, *η*^2^_p_ = 0.71, and between Color and Response, *F*(1,77) = 5.85, *p* < 0.05, *η*^2^_p_ = 0.07: repeating one but not the other feature slowed down responses; see Table 2. The remaining interaction was not significant, *F* < 1, *p* ≥ 0.89. From RTs Response × Shape (*M* = 43 ms, SD = 28) and Response × Color (*M* = 5 ms, SD = 19) stimulus–response binding effects were extracted. SDs in this study were comparable to other studies with bigger sample size (Colzato, Zmigrod & Hommel, 2013). Internal reliability of Shape–Color and Shape–Response was good to high, rSB1 = 0.257 and rSB1 = 0.636, respectively. However, the internal reliability of Color–Response binding was negative, rSB1 = − 0.303, which violates the reliability model assumptions.

**Table 2:** Mean RTs and PEs for responses to R2 as a function of the relationship between the responses (R1 and R2), and the relationship between the stimuli features (S1 and S2) for shape and color

Stimulus feature repeated	Response			
	Repeated		Alternated	
	RT	PE	RT	PE
Shape (S)	511 (111)	1.9 (3.4)	549 (110)	5.4 (5.8)
Color (C)	545 (115)	4.5 (6.7)	507 (96)	2.0 (4.4)
SC	493 (105)	1.2 (2.8)	541 (112)	6.0 (6.4)
Neither	546 (117)	5.3 (6.6)	497 (104)	1.3 (3.3)

Standard deviations (SD) of the mean are shown in parentheses

PEs revealed significant interactions between Shape and Response, *F*(1,77) = 74.68, *p* < 0.001, *η*^2^_p_ = 0.49, and Color and Response, *F*(1,77) = 7.02, *p* < 0.05, *η*^2^_p_ = 0.08. That is, repeating one but not the other feature elicited less accurate responses for shape–response and color–response bindings; see Table 2. All the remaining interactions were not significant either, *F*s < 1, *p*s ≥ 0.83.

### Attentional set-shifting task

The affect grid yielded the following scores: mood (*M* = 1.19; SD = 1.70) and arousal (*M* = − 0.14; SD = 1.80). Table 3 provides an overview of the relevant ANOVA outcomes for RTs and PEs. This analysis yielded a significant main effect for compatibility, *F*(1,77) = 111.50, *p* < 0.001, *η*2p = 0.59 (RTs): compatible trials were generally faster than incompatible trials. SDs in this study were comparable to other studies with the same sample size (Dreisbach et al., 2005). The internal reliability of the Switch Perseveration and Switch Learned Irrelevance was as follows: rSB1 = 0.390 and rSB1 = 0.131. Further, compatibility did not interact with transfer condition (*p* > 0.16). Error rate analysis did not yield any significant effects.

**Table 3:** Mean RTs and PEs for pre- and post-switch trials

Switch condition							
Perseveration				Learned Irrelevance			
Pre		Post		Pre		Post	
RT	PE	RT	PE	RT	PE	RT	PE
613 (102)	7.3 (8.9)	721 (133)	3.6 (6.8)	627 (120)	9.2 (10.6)	736 (133)	5.5 (8.5)

Standard deviations (SD) of the mean are shown in parentheses

### ANT

Before starting the ANT task, the affect grid revealed the following scores: mood (*M* = 1.67; SD = 1.58) and arousal (*M* = 0.28; SD = 1.76). Table 4 provides an overview of the relevant ANOVA outcomes for RTs and PEs. RTs and PEs revealed main effects of cue type, *F*(3,231) = 474.33, *p* < 0.001, *η*2p = 0.86 (RTs); *F*(3,231) = 3.99, *p* < 0.05, *η*2p = 0.05 (PEs), flanker congruency, *F*(2,154) = 535.89, *p* < 0.001, *η*2p = 0.87 (RTs); *F*(2,154) = 88.27, *p* < 0.001, *η*2p = 0.53 (PEs), and a significant interaction between flanker and cue type, *F*(6,462) = 4.97, *p* < 0.001, *η*2p = 0.06 (only for RTs), which indicates that spatially uninformative cues (central or double cue) lead to greater flanker interference effects than either no cue or spatially informative cues. From RTs alerting (no cue–double cue; *M* = 45 ms, SD = 26), orienting (center cue–spatial cue; *M* = 81 ms, SD = 31) and executive control (incongruent flanker–neutral flanker; *M* = 106 ms, SD = 40) effects were extracted. SDs in this study were comparable to other studies with the same sample size (Geva et al., 2013). Internal reliability of alerting, orienting, and executive control were, respectively, rSB1 = 0.209, rSB1 = 0.556, and rSB1 = 0.831.

**Table 4:** Mean (SD) RTs and PEs by flanker congruency and cue type

	Cue type			
	None	Center	Double	Spatial
RTs				
Incongruent	712 (87)	683 (90)	667 (87)	585 (91)
Congruent	610 (67)	562 (61)	559 (64)	488 (70)
Neutral	592 (64)	553 (61)	550 (62)	480 (68)
PEs				
Incongruent	9.34 (11.1)	8.60 (8.5)	7.42 (8.1)	6.19 (7.5)
Congruent	1.82 (6.6)	0.85 (2.4)	1.28 (2.9)	0.75 (1.7)
Neutral	2.51 (7.4)	1.71 (2.6)	1.49 (2.6)	1.71 (2.6)

### Correlations

#### Stroop task

The size of the Stroop effect correlated positively with both the rumination subscales in the LEIDS-R total score: higher rumination scores were associated with a more pronounced Stroop effect, see Table 5. These effects decreased and were no longer significant, after controlling for depressive symptoms and current mood and arousal: rumination (*r* = 0.162; *p* = 0.174), LEIDS-R total scores (*r* = 0.155; *p* = 0.193).

**Table 5:** Correlations between stimulus–response conflict cost in the Stroop task (as reflected by the congruency effect), event-file task (as indexed by the Color × Response and Shape × Response stimulus–response bindings), the attentional set-shifting task (as reflected by the switch perseveretion costs, switch learned irrelevance costs, and the difference between those costs), the ANT (as indexed by the alerting, orienting and conflict effects), and cognitive reactivity to sad mood (as indicated by LEIDS-r total score, and rumination and aggression subscales)

TASK			Stroop effect	Col × Res	Sh × Res	Switch persever	Switch learned irrelevance	Difference in switch costs	EC	Alerting	Orienting	LEIDS_Rum	LEIDS_Agg	LEIDS_ Tot
Stroop	Stroop effect	Pearson’s r	1	− 0.058	0.192	0.113	0.175	0.062	0.062	0.065	0.007	0.244*	0.187	0.247*
		*p* value		0.617	0.097	0.331	0.131	0.592	0.595	0.575	0.954	0.034	0.105	0.032
Event-File	Col × Res	Pearson’s r		1	− 0.300**	− 0.060	0.070	0.105	− 0.018	0.041	0.078	0.332**	0.262*	310**
		*p* value			0.008	0.602	0.541	0.360	0.872	0.722	0.495	0.003	0.020	0.006
	Sh × Res	Pearson’s r			1	0.182	0.204	0.035	0.005	− 0.022	− 0.138	0.010	− 0.093	0.018
		*p* value				0.111	0.073	0.758	0.966	0.850	0.227	0.930	0.419	0.877
Att. set-shifting	Switch persever	Pearson’s r				1	0.227*	− 565***	− 0.026	− 0.036	− 0.132	− 0.013	0.053	− 0.005
		*p* value					0.046	0.0001	0.818	0.756	0.249	0.911	0.643	0.968
	Switch learned irrelevan	Pearson’s r					1	675***	− 0.100	0.084	0.056	− 0.080	− 0.94	− 0.099
		*p* value						0.0001	0.386	0.467	0.626	0.486	0.411	0.388
	Diff switch costs	Pearson’s r						1	− 0.064	0.098	0.148	− 0.058	− 0.120	− 0.080
		*p* value							0.575	0.394	0.197	0.613	0.294	0.484
ANT	EC	Pearson’s r							1	− 0.043	− 0.150	0.079	0.098	0.088
		*p* value								0.710	0.191	0.491	0.394	0.444
	Alerting	Pearson’s r								1	− 0.210	− 0.089	− 0.053	− 0.148
		*p* value									0.065	0.436	0.642	0.197
	Orient	Pearson’s r									1	0.028	− 0.004	− 0.069
		*p* value										0.811	0.973	0.547
Cognitive reactivity to sad mood	LEIDS_Rum	Pearson’s r										1	0.377**	0.804***
		*p* value											0.001	0.0001
	LEIDS_Agg	Pearson’s r											1	0.622***
		*p* value												0.0001
	LEIDS_Total	Pearson’s r												1
		*p* value												

*p < 0.05, **p < 0.01, ***p < 0.001

#### Event-file task

The size of the color–response binding effect correlated positively with all three scores of the LEIDS-R: as expected, higher rumination scores were associated with a more pronounced effect of the retrieval of the task-irrelevant stimulus–response feature binding; see Table 5. These effects remained stable and significant after controlling for depressive symptoms and current mood and arousal: LEIDS-R total scores (*r* = 0.313; *p* = 0.007), rumination (*r* = 0.316; *p* = 0.006), and aggression (*r* = 0.236; *p* = 0.043). Also of interest, the effect of the binding between the task-relevant stimulus shape and the response was far from engaging in any statistically relevant relationship with any of the scales.

#### Attentional set-shifting task

Three different scores were considered: the switching costs for the perseveration condition, the switching costs for the learned irrelevant condition, and the difference between these two—a score that represents the interaction between compatibility and condition. However, none of these scores correlated significantly with any of the LEIDS-R scores.

#### ANT

No significant correlations were obtained between LEIDS-R total scores or any subscale and the alerting, orienting, and executive control effects of the ANT; see Table 5.

#### Bayesian Pearson correlations

A Bayesian correlational analysis (using the default setting) was carried out to quantify evidence for the presence of a cognitive reactivity to sad mood effect (BF_10_) on our nine key task indicators (and, as a control, the interaction between stimulus shape and response in the event-file task); see Table 6. A BF_10_ larger than 1 indicates that the data are more likely to occur under H1 than under H0. Only the correlation between the size of the color–response binding effect and LEIDS-R rumination score received strong evidence for H_1_. All other key task indicators received no evidence or only anecdotal evidence for H_0_ or H_1_.

**Table 6:** Bayesian Pearson correlations between stimulus–response conflict cost in the Stroop task (as reflected by the congruency effect), event-file task (as indexed by the Color × Response and Shape × Response stimulus–response bindings), the attentional set-shifting task (as reflected by the switch perseveretion costs, switch learned irrelevance costs, and the difference between those costs), the ANT (as indexed by the alerting, orienting, and conflict effects), and cognitive reactivity to sad mood (as indicated by LEIDS-r total score, and rumination and aggression subscales)

TASK			Stroop effect	Col × Res	Sh × Res	Switch persever	Switch learned irrelevance	Difference in switch costs	EC	Alerting	Orienting	LEIDS_Rum	LEIDS_Agg	LEIDS_ Tot
Stroop	Stroop effect	Pearson’s r	1	− 0.058	0.192	0.113	0.175	0.062	0.062	0.065	0.007	0.244*	0.187	0.247*
		BF_10_		0.162	0.553	0.228	0.439	0.165	0.165	0.167	0.144	1.303	0.520	1.380
Event-File	Col × Res	Pearson’s r		1	− 0.300**	− 0.060	0.070	0.105	− 0.018	0.041	0.078	0.332*	0.262*	0.310**
		BF_10_			4.681	0.162	0.170	0.213	0.143	0.151	0.178	10.843	1.974	6.047
	Sh × Res	Pearson’s r			1	0.182	0.204	0.035	0.005	− 0.022	− 0.138	0.010	− 0.093	0.018
		BF_10_				0.492	0.687	0.148	0.142	0.144	0.289	0.142	0.195	0.143
Att. set-shifting	Switch persever	Pearson’s r				1	0.227*	− 0.565***	− 0.026	− 0.036	− 0.132	− 0.013	0.053	− 0.005
		BF_10_					1.006	214735.005	0.145	0.148	0.272	0.142	0.157	0.142
	Switch learned irrelevan	Pearson’s r					1	0.675***	− 0.100	0.084	0.056	− 0.089	− 0.94	− 0.099
		BF_10_						8.939e+8	0.205	0.183	0.159	0.180	0.197	0.204
	Diff switch costs	Pearson’s r						1	− 0.064	0.098	0.148	− 0.058	− 0.120	− 0.080
		BF_10_							0.165	0.202	0.320	0.160	0.243	0.180
ANT	EC	Pearson’s r							1	− 0.043	− 0.150	0.079	0.098	0.088
		BF_10_								0.151	0.327	0.179	0.202	0.189
	Alerting	Pearson’s r								1	− 0.210	− 0.089	− 0.053	− 0.148
		BF_10_									0.754	0.191	0.157	0.321
	Orient	Pearson’s r									1	0.028	− 0.004	− 0.069
		BF_10_										0.146	0.142	0.169
Cognitive reactivity to sad mood	LEIDS_Rum	Pearson’s r										1	0.377**	0.804***
		BF_10_											41.463	7.832e+15
	LEIDS_Agg	Pearson’s r											1	0.622***
		BF_10_												1.030e+7
	LEIDS_Total	Pearson’s r												1
		BF_10_												

*p < 0.05, **p < 0.01, ***p < 0.001

## Discussion

The aim of the study was to throw more light on the relationship between rumination and cognitive-control processes. For that purpose, we tried to replicate the previously observed positive relationship between rumination tendencies in the Stroop effect and added eight further indicators of performance in theoretically relevant tasks or task aspects. With regard to the Stroop effect, we made two interesting observations. For one, our standard analysis replicated the previous findings of Philippot and Brutoux (2008) that more rumination goes with a more pronounced Stroop effect. At the same time, however, we also found that controlling for depressive tendencies eliminated this effect and that analysis within the Bayesian framework found no evidence in supporting H_0_ or H_1_. This means that with respect to the Stroop effect, our data are inconclusive in providing an account for the previous failures to replicate the relationship between rumination and Stroop (Philippot & Brutoux, 2008; Krompinger & Simons, 2011; Meiran et al., 2011).

Less sensitive to self-reported depression symptoms was our second measure, the degree to which previous bindings between irrelevant stimulus information and the response was retrieved in the next trial. The corresponding color-by-response effect strongly correlated with all three scales irrespective of depressive tendencies, but we found moderate evidence for H_1_ only for the rumination score. This provides reasonable evidence that rumination is related to the control of retrieving internal information and, in particular, to preventing irrelevant information from being retrieved. Nevertheless, given that the correlation of rumination scores with the effect of the binding between the relevant stimulus feature and the response and of the correlation between rumination and the three ANT scores received no evidence or only anecdotal evidence for H0 or H1, it is difficult to say how specific the impact of rumination is to the control of retrieving internal information. Supported by Bayesian inference, future studies need to search for converging evidence that rumination is associated with impairments of the handling of distractor information (Whitmer & Gotlib, 2013) and for the idea that it is not the mere presence of an external distractor that ruminating individuals find difficult to deal with, but the handling of information that such distractors may activate in one’s memory.

Our conclusion is that rumination reliably affects the efficiency of memory-retrieval control in the face of events that activate irrelevant memory information. This allows the prediction that rumination might be beneficial in tasks that require or that benefit from less selective memory retrieval. In other words, the fact that a greater tendency to ruminate was associated with less efficient processing in the present study might be taken as a reflection of the choice of tasks rather than a demonstration of a generalized deficit associated with rumination.

### Footnote 1

As standard procedure in our laboratory, at the start of each session, participants completed a visual analog scale (range of scores from 0 to 100) that measured the subjective self-reported current level of anxiety, nervousness, insecurity, and stress. Next, heart rate (HR) data were measured for 5 min using a Polar H7 heart rate monitoring system (Polar Electro, Kempele, Finland), which wirelessly receives HR data from a chest strap worn by the participants. As a result of technical problems, several data were lost across all five sessions. Further, in the first session participants were weighed and their BMI was measured using an OMRON Body Composition Scale Karada Scan. Moreover, their daily level of physical activity and smoking behavior were recorded.
